# Flame Pyrolysis Synthesis of Mixed Oxides for Glycerol Steam Reforming

**DOI:** 10.3390/ma14030652

**Published:** 2021-01-31

**Authors:** Francesco Conte, Serena Esposito, Vladimiro Dal Santo, Alessandro Di Michele, Gianguido Ramis, Ilenia Rossetti

**Affiliations:** 1 Chemical Plants and Industrial Chemistry Group, Dip. Chimica, Università degli Studi di Milano, INSTM Unit Milano-Università and CNR-SCITEC, Via C. Golgi, 19, 20133 Milano, Italy; francesco.conte@unimi.it (F.C.); ilenia.rossetti@unimi.it (I.R.); 2 Dipartimento di Scienza Applicata e Tecnologia e Unità INSTM Torino-Politecnico Corso Duca degli Abruzzi, 24, 10129 Torino, Italy; 3 CNR-Istituto di Scienze e Tecnologie Chimiche “Giulio Natta”, Via C. Golgi 19, 20133 Milano, Italy; vladimiro.dalsanto@scitec.cnr.it; 4 Dip. Fisica e Geologia, Università degli Studi di Perugia, Via Pascoli, 06123 Perugia, Italy; alessandro.dimichele@collaboratori.unipg.it; 5 Dip. di Ingegneria Civile, Chimica e Ambientale, Università degli Studi di Genova and INSTM Unit Genova, Via all’Opera Pia 15A, 16145 Genoa, Italy; gianguidoramis@unige.it

**Keywords:** glycerol steam reforming, H_2_ production, flame spray pyrolysis, Ni mixed oxide catalysts, coking

## Abstract

Flame spray pyrolysis was used to produce nanosized Ni-based catalysts starting from different mixed oxides. LaNiO_3_ and CeNiO_3_ were used as base materials and the formulation was varied by mixing them or incorporating variable amounts of ZrO_2_ or SrO during the synthesis. The catalysts were tested for the steam reforming of glycerol. One of the key problems for this application is the resistance to deactivation by sintering and coking, which may be increased by (1) improving Ni dispersion through the production of a Ni-La or Ni-Ce mixed oxide precursor, and then reduced; (2) using an oxide as ZrO_2_, which established a strong interaction with Ni and possesses high thermal resistance; (3) decreasing the surface acidity of ZrO_2_ through a basic promoter/support, such as La_2_O_3_; and (4) adding a promoter/support with very high oxygen mobility such as CeO_2_. A further key feature is the use of a high temperature synthesis, such as flame spray pyrolysis, to improve the overall thermal resistance of the oxides. These strategies proved effective to obtain active and stable catalysts at least for 20 h on stream with very limited coke formation.

## 1. Introduction

**Figure 1:**
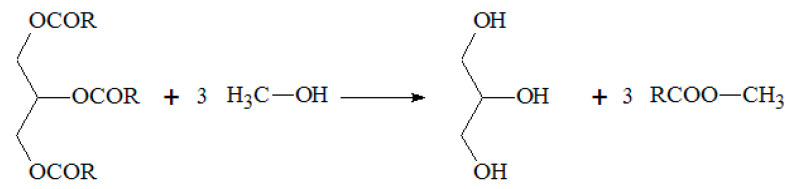
Reaction equation of the transesterification process.

Hydrogen is gaining increasing importance as an energy vector, as a means to store energy and to convert it through highly efficient devices, such as fuel cells. However, H_2_ is not present as such in nature and it has to be produced [1,2]. Industrially, it is mainly obtained from the steam reforming of hydrocarbons, but currently, the research is attempting to adapt this process to renewable sources [3]. Among these, bioethanol, coming from the fermentation of biomasses, and glycerol, a by-product from the trans-esterification of triglycerides for biodiesel production (Figure 1), are among the most studied [4,5].

The current worldwide production of biodiesel is ca. 702 thousand barrels/day [6]. The reaction leaves ca. 10 wt% glycerol as a byproduct per unit weight of biodiesel produced [7,8,9,10]. However, crude glycerol contains mineral impurities (phosphorous, calcium, alkali metals, unreacted fatty acids and methanol), which impose expensive purification to achieve practically exploitable purity for the conventional glycerol market (food and pharma industry). Furthermore, the latter market is unable to sustain the increasing availability of glycerol. The development of glycerol transformation routes that have a large potential volume of production and do not require heavy purification of the feed is envisaged and, among these, the production of hydrogen by steam reforming appears promising [11].

Glycerol steam reforming (GSR) occurs through the following stoichiometry:

C_3_H_8_O_3_ + 3 H_2_O → 3 CO_2_ + 7 H_2_   ∆H = +128 kJ/mol

which may be seen as the combination of syngas production and the water gas shift reaction:

C_3_H_8_O_3_ → 3 CO + 4 H_2_    ∆H = +251 kJ/mol

CO + H_2_O ⇆ CO_2_ + H_2_     ∆H = −41 kJ/mol

Undesired parasitic reactions may also occur, among which those leading to carbon deposition are of particular concern.

Hydrogen yield depends on operating conditions, the nature of active metal, and the catalyst support. The most studied catalysts for steam reforming of glycerol are based on noble (Rh, Ru, Pt, Pd) and non noble (Ni, Cu) metals. Among them, Ni catalyst is the most attractive for its own ability in the cleavage of the C-C, C-H, and H-O bonds [12,13,14,15,16,17,18,19]. Besides, it is cost-effective, but suffers deactivation in long-term operations due to coke formation and metal sintering [20]. Therefore, synthesis of suitable catalysts is addressed towards the suppression of coke formation and sintering of the metal particles during reforming. Nowadays, these limitations are not yet overcome and the preparation of a stable catalyst for glycerol steam reforming is still a challenge.

Different approaches have been employed to improve the performance of nickel-based catalysts, most of them based on tailoring the physical properties. High surface area with suitable nickel dispersion along with small particle size and a strong metal–support interaction are seen as the key factors for the synthesis of highly active catalysts. From this point of view, supports play an important role in terms of hydrogen selectivity and catalyst stability.

Alumina is one of the most used supports for its mechanical and physical properties. Lima et al. [12] studied the effect of Mg addition to decrease the acid character of alumina reported to favor coke formation. The presence of Mg also led to a better Ni dispersion through the formation of a spinel structure, Mg(Ni)Al_2_O_4_. The authors also demonstrated the non-negligible influence of the preparation method by comparing impregnated and co-precipitated samples: the partial replacement of Ni with Mg had a greater effect on the co-precipitated samples with respect to impregnated catalysts.

A different approach was proposed by Yancheshmeh et al. [13], who exploited the activity of NiAl_2_O_4_-based catalysts. The better dispersion achieved embedding Ni in a spinel structure encounters the difficulty of a limit reduction with respect to a supported NiO. To overcome this limitation, the authors investigated the effect of a novel solvothermal method achieving high H_2_ yields (76%) and 95% glycerol conversion into gaseous products.

Another promising candidate as catalytic support is ZrO_2_ in name of its thermal stability and surface properties [21]. The effect of the crystalline polymorphs on the steam reforming of glycerol was studied by Dahdah et al. [14]; nickel was impregnated on synthesized tetragonal zirconia and on commercial monoclinic zirconia. Ni-(monoclinic)zirconia showed enhanced nickel reducibility, which positively affected hydrogen yields and glycerol conversion.

The replacement in crystalline lattice of Zr^4+^ with lower valence cations (Mg^2+^, Ca^2+^, Y^3+^, La^3+^) produces oxygen vacancies [22,23]. The concentration of oxygen defects and the extent of oxygen mobility are crucial in many applications of doped zirconia. Charisiou et al. [15] tested yttria-stabilized zirconia as catalytic support in the GSR process. Y_2_O_3_ modified ZrO_2_ led to more reducible NiO, less sintering phenomena, and higher stability. Moreover, whereas graphitic structures are identified on unpromoted zirconia, amorphous carbon was deposited on Ni/YZr, which are more easily oxidized during the reaction. The effect of a highly defective La_2_O_3_-ZrO_2_ support was also tested by Veiga et al. [16]. Here, 15% Ni-La-Zr was used as catalyst for GSR using a feedstock of glycerol obtained directly from an industrial biodiesel plant as a by-product. The authors observed a strong correlation between the calcination temperature and the amount of carbon deposits. The lowest amount of carbon deposits for the catalyst calcined at 850 °C was correlated with the formation of a pyrochlore-type structured La_2_Zr_2_O_7_ with a partial substitution of nickel ions into Zr sites. The presence of defects and the subsequent formation of mobile oxygen vacancies favored the oxidation of carbon deposits during the reaction.

The effects of various supports, different promoters (K, Ca, Sr, Ce, La, Cr, Fe), and process conditions were, recently, illustrated by a review of Saeidabad et al. [17]. Among the different catalytic formulations, perovskite type oxide appears intriguing for their unique crystal structure, which promotes the formation of metallic particles with high dispersion, enhanced hydrogen production, and improved coke resistance.

In addition to the structural and physicochemical properties of the support, the adopted synthesis approach is also a powerful tool for obtaining highly active and stable catalysts. A valuable and intriguing synthesis route is represented by the use of metal–organic frameworks as precursors of metal oxides-based materials [24,25,26].

Among the possible active phases, Pt and other noble metals are effective for this reaction and are used for aqueous phase reforming, but they are prone to deactivation and expensive. Ni offers the advantage of a reasonable activity and selectivity, coupled with smaller cost with respect to noble metals for this application. However, Ni readily forms carbon nanotubes from carbide intermediates and we have previously demonstrated that, during the steam reforming of bioethanol, this may lead to the detachment of Ni particles, with irreversible catalyst deactivation [27,28,29,30,31,32,33,34,35,36,37,38]. We have already demonstrated that a strong metal–support interaction also helps keep Ni well dispersed under the working conditions for the steam reforming of bioethanol.

Acidic supports such as alumina, the most used for methane steam reforming, are more prone to coking for this application, as dehydration and condensation may occur over the acidic sites. Therefore, different strategies may be developed to limit this phenomenon, e.g., the addition of basic oxides (Mg, La, Ce, Zr) [20,39,40] or the use of supports intrinsically characterised by high oxygen mobility, such as CeO_2_ or Ce_1−x_Zr_x_O_2_. 

In order to improve the dispersion of Ni over these oxides, mixed oxide formulations can be used, such as La_1−x_Ce_x_NiO_3_ (with x = 0, 0.1, 0.3, 0.7), to achieve a suitable activity and resistance, at least for a limited time-on-stream [41,42,43].

Therefore, in the present work, we have prepared and characterised a set of Ni-based catalysts supported over mixed oxides. The selected synthesis procedure was flame spray pyrolysis (FP), which should guarantee suitable thermal resistance thanks to the high temperature reached during the synthesis. The Ni active phase was added directly during the flame preparation, possibly in form of mixed oxide, and then reduced. The FP method already proved effective for the steam reforming of bioethanol to improve the ni dispersion and therma resistance for a set of LaNiO_3_ samples [44]. Alternatively, we tested the impregnation of Ni over the same supports prepared by FP. The performance and resistance to deactivation were tested for the steam reforming of glycerol.

## 2. Experimental

### 2.1. Materials’ Preparation

The catalysts were prepared by flame spray pyrolysis (FP) [44,45]. The precursors of the oxide catalysts were dissolved with 0.1 M concentration in a 1:1 (vol/vol) solution of propionic acid pur. 97% (Aldrich-Merck Life Science S.r.l., Italy) and o-xylene pur. 97% (Aldrich-Merck Life Science S.r.l., Italy), acting as a fuel for the flame and the solution injected through a syringe pump (Harvard, mod. 975, Harvard Apparatus, Holliston, MA, USA) through a needle with a liquid flow of 2.2 mL/min. The needle is inserted in a self-designed burner, where it is surrounded by a coaxial flow of 5 NL/min of O_2_, with a 1.5 bar pressure drop across the nozzle, and the mixture is ignited by a ring of flamelets (CH_4_ and O_2_ with flowrate of 0.5 and 1 L/min, respectively). Gas flows are regulated by means of MKS, mod. 1259 massflowmeters (MKS Instruments, Inc., Andover, MA, USA). The powder is collected on a pyrex bell surrounding the burner.

The precursors used were as follows: La(CH_3_COO)_3_·H_2_O pur. 99.9% (Aldrich-Merck Life Science S.r.l., Italy), Ni(CH_3_COO)_2_·4H_2_O pur. 98% (Aldrich-Merck Life Science S.r.l., Italy), Zr(C_5_H_7_O_2_)_4_ pur. 99% (Aldric-hMerck Life Science S.r.l., Italy), Sr(CH_3_COO)_2_ pur. 99.9% (Aldrich-Merck Life Science S.r.l., Italy), and Ce(CH_3_COO)_3_·H_2_O pur. 99.9% (Aldrich-Merck Life Science S.r.l., Italy).

The catalyst formulations prepared are reported in Table 1. The samples with the symbol 10 wt% Ni/Oxide were prepared by impregnation of the metal, from the same precursor, over the FP-synthesised oxide, used as support. Here, 10 wt% metal loading was selected from a previous investigation [28]. The formulations where Ni is included in the oxide formula were prepared as mixed oxides. The metallic Ni active phase was achieved upon reduction in the activation step.

### 2.2. Catalysts’ Characterisation

The catalysts (0.1 g) were characterised by temperature programmed reduction (TPR) with a Micromeritics Pulse Chemisorb 2700 (Norcross, GA) with TCD (Thermo-Conductivity) detector, by heating 8 °C/min up to 950 °C. The catalysts were pretreated in oxygen flow at 250 °C and then analysed in 20 mL/min of a 5 vol% H_2_/Ar mixture.

The specific surface area of the catalysts was determined with by N_2_ adsorption/desorption with a Micromeritics ASAP2010 instrument (Norcross, GA) after outgassing the sample overnigth at 300 °C.

The catalysts were characterized by X-ray diffraction (XRD) on a Philips PW3020 powder diffractometer (Philips, Eindhoven, NL).

Morphologic analysis, obtained by Scanning Electron Microscopy (SEM) was carried out with an Electron Scanning Microscope Philips XL-30CP (Philips, Eindhoven, NL). Elemental composition was determined using the Energy dispersive X-ray analysis (EDS) with an EDAX/DX4 detector.

Trasmission Electron Microscopy (TEM) images were obtained using a Philips 208 Transmission Electron Microscope (Philips, Eindhoven, NL). The samples were prepared by putting one drop of an ethanol dispersion of the catalysts on a copper grid pre-coated with a Formvar film and dried in air.

Thermal Gravimetric Analysis (TGA) analysis was carried out on 10 mg of spent sample (resolution 0.001 mg) with a Perkin Elmer TGA7 apparatus (Perkin Elmer Italia, Milano, Italy), by heating 2 °C/min from 50 to 1000 °C to quantify the amount of carbon residuals over the catalyst.

### 2.3. Catalytic Activity Testing

Activity testing was performed in a quartz tubular reactor, with 0.9 cm inner diameter, loaded with 0.200 g of catalyst, diluted 1:1 (wt/wt) with quartz of the same size (354–500 µm). The catalyst bed is separated by quartz wool from the top fillers, where, in the evaporative zone, metal beads are used (1.2–4.8 mm). The reactor is heated by an electric oven, controlled by a Eurotherm mod. 2408 (Eurotherm, Worthing, UK) TIC, Temperature Indicator and Controller, for the reactive zone, while the upper evaporative zone is heated separately with an Eurotherm mod. 808 (Eurotherm, Worthing, UK ) TIC. The reactor is connected with gas lines that can feed either He (30 mL/min during activity testing) or H_2_. A 10 wt% aqueous solution of glycerol is pumped with a KNF-Lab Stepdos 03S (KNF, Stockholm, Sweden) with a flow rate of 0.060 mL/min. The liquid products are condensed at the reactor outlet through a cryogenic bath (1.5 °C), while the gaseous products pass through a flowmeter (Bios 530 L) and then are directly fed to a Gas Chromatograph (Agilent mod. 6890N, Palo Alto, CA, USA) for periodic samplings.

The catalyst is activated in 30 mL/min of H_2_ by heating by 10 °C/min up to 700 °C, and kept for 1 h. The temperature is then decreased to 650 °C in the reaction zone, while the evaporating zone is maintained at 250 °C. The tests were typically carried out for 20 h on stream, if not specified otherwise. The results report glycerol conversion; hydrogen yield; and the selectivities to CO, CO_2_, and CH_4_ if relevant. When needed, the condensed products were analysed with a GC-MS Hewlett Packard 5971 Series (Agilent, Palo Alto, CA, USA) at the end of the reaction. Replicates were reproducible within 5% error.

## 3. Results and Discussion

### 3.1. Catalysts’ Characterisation

The specific surface area of the catalysts was between 44 and 63 m^2^/g (Table 1), with very limited micropores contribution, because the FP procedure leads to the formation of dense nanoparticles.

**Figure 2:**
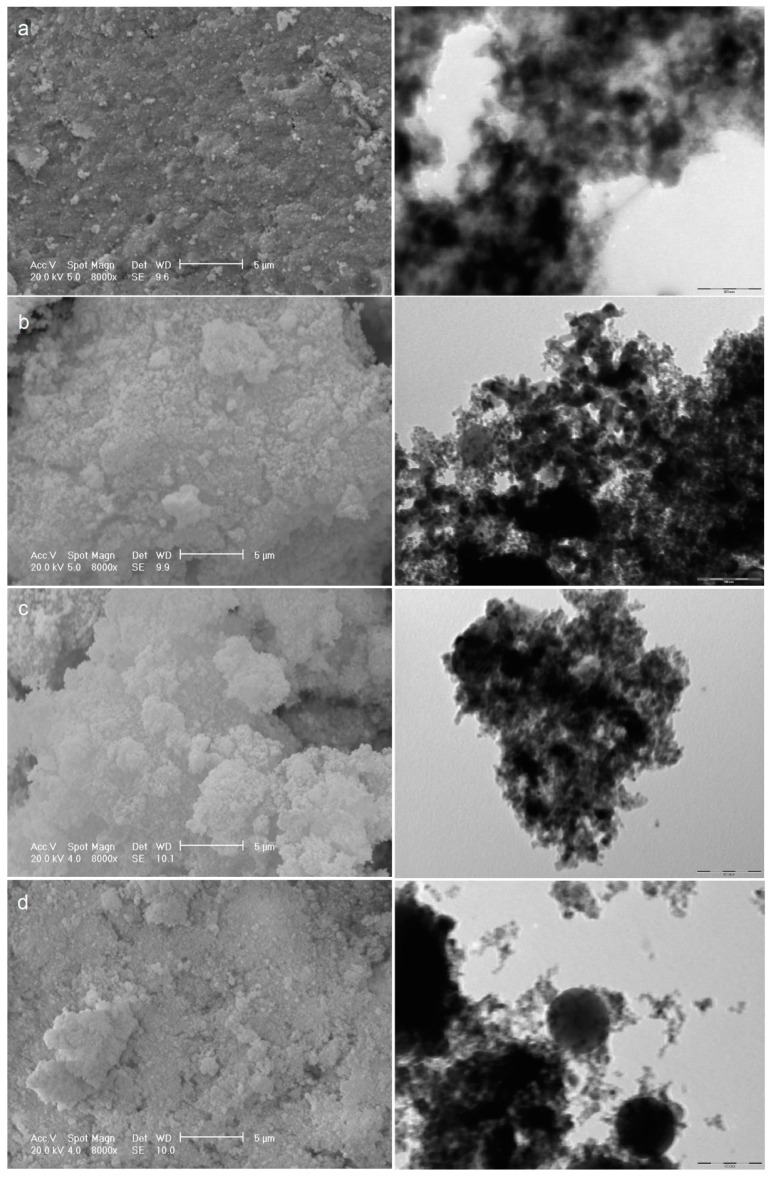
SEM (left, marker size 5 µm) and TEM (right, marker size 100 nm) micrographs of selected samples (**a**) La_0.3_Zr_0.7_NiO_3_; (**b**) La_0.3_Ce_0.7_O_3_; (**c**) La_0.8_Sr_0.2_NiO_3_; (**d**) 10 wt% Ni/(0.3La_2_O_3_–0.7ZrO_2_).

SEM and TEM analysis revealed uniform nanoparticles with size lower than 20 nm (Figure 2). Some bigger agglomerates were visible only for sample 10 wt% Ni/(0.3La_2_O_3_–0.7ZrO_2_), for which bigger hollow spheres were evident in some TEM pictures (Figure 2).

The nominal composition of the samples was checked through EDS analysis, considering the atomic% (at%). Mapping the sample surface revealed quite uniform distribution of all the elements over the surface of the catalyst. When considering spot analysis, the average composition of the samples was in general confirmed.

For instance, for sample La_0.3_Ce_0.7_NiO_3_ (La = 6.61 at%, Ce = 15.36 at%, Ni = 21.39 at%), the atomic ratio La/(La + Ce) was 0.301, while (La + Ce)/Ni was 1.03. Similarly, for sample La_0.8_Sr_0.2_NiO_3_ (La = 21.4 at%, Sr = 5.1 at%, Ni = 23.7 at%), the atomic ratios were Sr/(Sr + La) = 0.19 and (La + Sr)/Ni = 1.12.

On the contrary, the Zr-containing samples revealed an unbalanced composition of the oxide, as the mixed phase was not formed and phase segregation occurred (see XRD results), resulting in a local composition different from the nominal one. For instance, the EDS analysis of sample 10 wt% Ni/(0.3La_2_O_3_–0.7ZrO_2_) (La = 5.8 at%, Zr = 70.7 at%,) revealed that the atomic ratio La/Zr was 0.08 instead of the nominal 0.42 and Ni content was 4.2 wt%. Similar results were evident for sample La_0.3_Zr_0.7_NiO_3_ (La = 4.4 at%, Zr = 52.5 at%, Ni = 17.6 at%), for which the La/Ni average atomic ratio was 0.25 instead of 0.3, but La/(La + Zr) was 0.08 with respect to the nominal 0.3.

**Figure 3:**
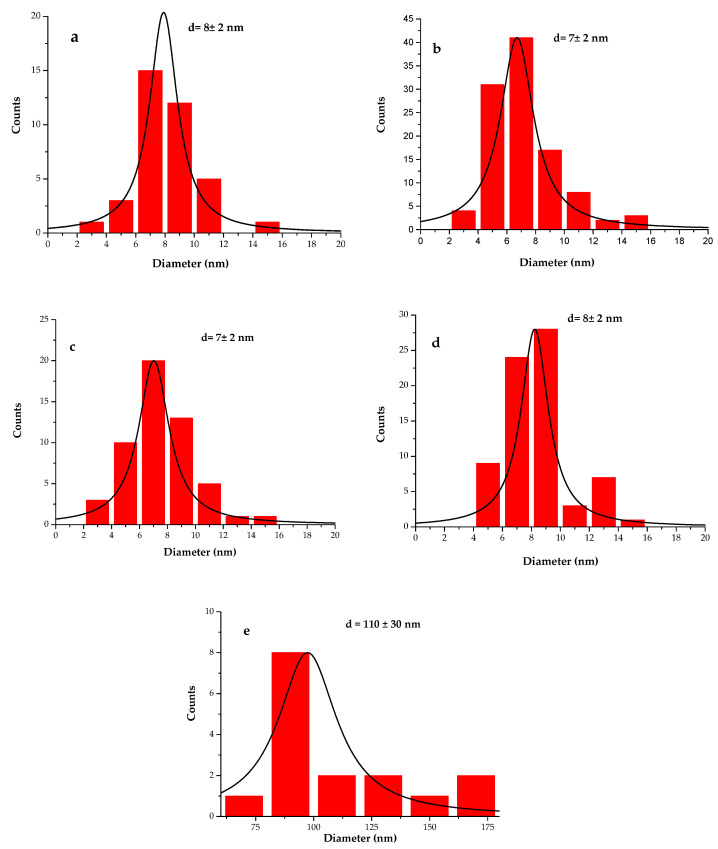
Particle size distributions of the selected samples (**a**) La_0.3_Zr_0.7_NiO_3_; (**b**) La_0.3_Ce_0.7_NiO_3_; (**c**) La_0.8_Sr_0.2_NiO_3_; (**d**) 10 wt%Ni(0.3La_2_O_3_–0.7ZrO_2_); (**e**) 10 wt%Ni(0.3La_2_O_3_–0.7ZrO_2_).

The particle size distribution statistics are reported in Figure 3. The average particle size is similar for all the samples and sufficiently sharp, except for sample 10 wt% Ni/(0.3La_2_O_3_–0.7ZrO_2_). The latter showed a narrow distribution centre as well on ca. 8 nm when excluding the bigger particles, while, when including the widest hollow particles, a broader distribution was obtained.

**Figure 4:**
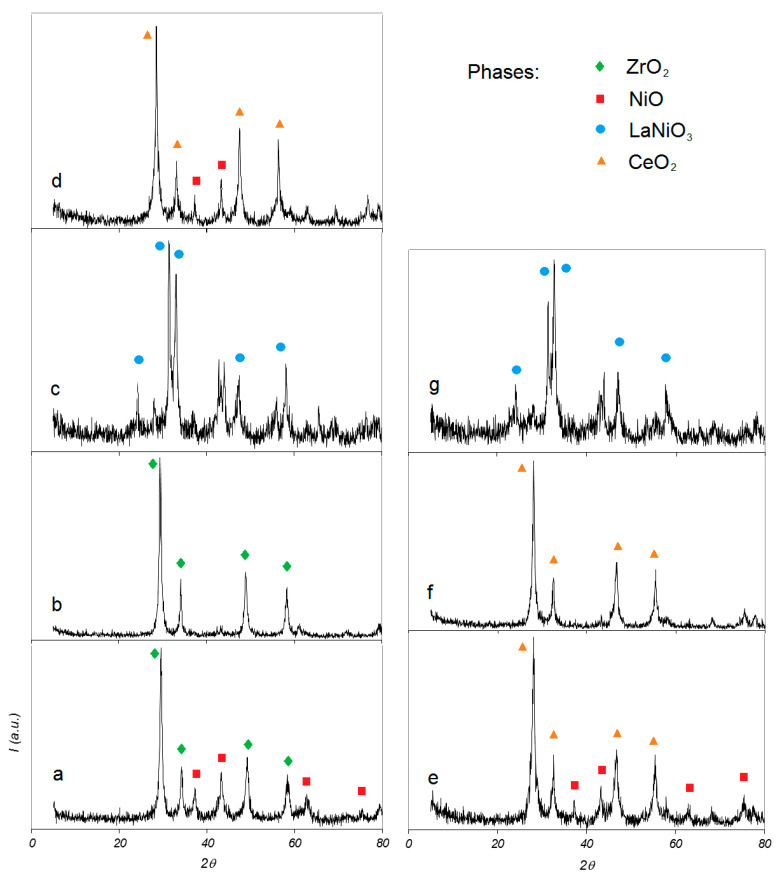
XRD patterns of the flame spray pyrolysis (FP)-prepared catalysts.(**a**) La_0.3_Zr_0.7_NiO_3_; (**b**) 10 wt% Ni/(0.3La_2_O_3_–0.7ZrO_2_); (**c**) La_0.8_Sr_0.2_NiO_3_; (**d**) CeNiO_3_; (**e**) La_0.3_Ce_0.7_NiO_3_; (**f**) 10 wt% Ni/(0.3La_2_O_3_–0.7CeO_2_); (**g**) LaNiO_3_.

The nature of the phases formed was investigated by XRD (Figure 4) and phase attribution was done through comparison with powder diffraction files (PDF, Centre for Diffraction Data, ICDD, charts [46]). The samples with formal formula La_0.3_Zr_0.7_NiO_3_ and 10 wt% Ni/(0.3La_2_O_3_–0.7ZrO_2_) were confirmed to be constituted of a heterogeneous phase mixture mainly containing ZrO_2_ and NiO. The latter phase was predominantly evident in the former sample owing to the higher Ni content than the latter sample, where Ni was added by impregnation in a lower amount.

Moreover, the samples with nominal formula CeNiO_3_, La_0.3_Ce_0.7_NiO_3_, and 10 wt% Ni/(0.3La_2_O_3_–0.7CeO_2_) showed the main reflection peaks of CeO_2_ and NiO, the latter hardly visible in the impregnated sample, due to the lower concentration. On the contrary, LaNiO_3_ and the same sample partially substituted with Sr revealed the typical reflections of the LaNiO_3_ phase. All the samples showed quite broad and poorly intense reflections, as a measure of the low crystal size and nanostructuring, typical of the FP synthesis method.

**Figure 5:**
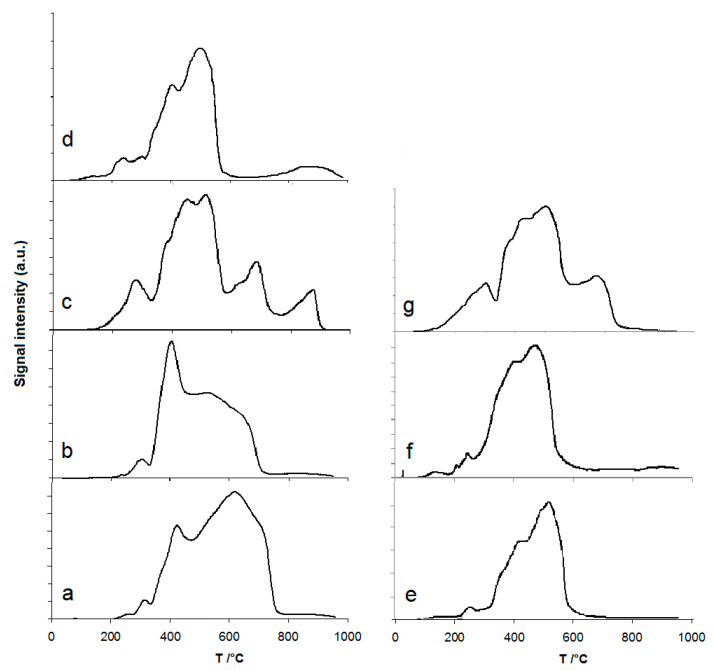
Temperature programmed reduction (TPR) patterns of the FP-prepared catalysts. Signal intensity in arbitrary units. (**a**) La_0.3_Zr_0.7_NiO_3_; (**b**) 10 wt% Ni/(0.3La_2_O_3_–0.7ZrO_2_); (**c**) La_0.8_Sr_0.2_NiO_3_; (**d**) CeNiO_3_; (**e**) La_0.3_Ce_0.7_NiO_3_; (**f**) 10 wt% Ni/(0.3La_2_O_3_–0.7CeO_2_); (**g**) LaNiO_3_.

Temperature programmed reduction (TPR, Figure 5) was carried out on the samples to determine the temperature range for the activation of the active phase, metallic Ni, in some samples available in the form of mixed oxide, and in other cases as NiO or as impregnated precursor salt. As already widely discussed elsewhere, the reduction temperature for the same oxide can be correlated to its dispersion and to the strength of the interaction with the support. The higher the reduction temperature, the stronger such interaction and typically the higher the metal oxide dispersion. Sharp reduction patterns also testify to the presence of homogeneous types of sites.

In the present samples NiO reduced between 200 and 600 °C, with broader reduction peaks when the supporting oxide was based on La/Zr than when supported over La/Ce (pattern *a* compared with *e* and pattern *b* compared with *f*). A reduction peak at high temperature (ca. 800 °C) appeared for CeNiO_3_ due to the partial reduction of the CeO_2_ support, while ZrO_2_ and La_2_O_3_ did not reduce under the present conditions.

The highest was the amount of NiO included as mixed phase into the support oxide, determined by XRD, the highest was the average reduction temperature of NiO. For instance, for the samples La_1−x_Sr_x_NiO_3_, three or four reduction peaks were present, with the highest temperature ones above 600 °C. For LaNiO_3_, curve *g*, differently reducible Ni^2+^ species were present and the predominant ones were reducing between 300 and 600 °C. By contrast, for sample La_0.3_Zr_0.7_NiO_3_ (curve *a*), the highest intensity peak appeared between 500 and 750 °C, underlining the presence of a higher concentration of less reducible Ni^2+^.

Furthermore, curves *d* and *e*, corresponding to similar Ce-containing samples, with or without La_2_O_3_, were practically overlapping, but with a very slight shift towards higher temperatures for the sample containing lanthanum oxide.

The direct incorporation of Ni during the synthesis led to a generaly higher reduction temperature (curves *a* vs. *b* for La-Zr and *e* vs. *f* for La-Ce) with respect to the samples obtained by impregnation of Ni, though maintaining a similar reduction profile.

Summing up, the highest Ni dispersion and strength of interaction with the support is shown for the La_1-x_Sr_x_NiO_3_ and La-Zr-Ni samples, especially when the latter are prepared as mixed oxides with Ni incorporation during the support synthesis. This, in princible, would ensure higher thermal resistance towards sintering and higher robustness towards the coking features. Indeed, coking implies carbides’ accumulation in the metal and overall leads to the formation of C nanotubes, as widely discussed in the literature [38,47,48]. This mechanism is inhibited when Ni is well dispersed and strongly interacting with the support.

### 3.2. Catalytic Activity

The catalysts were tested for the steam reforming of glycerol, focusing mainly on the characterisation of the gas phase products and on the stability with time-on-stream.

Reactant conversions were referred to glycerol, i.e., the limiting reactant, calculated as in Equation (1), where *in* represent the molar florates entering (in) or outflowing (out) the reactor. The selectivity to each detected product (Equation (2)) was calculated with respect to the reacted glycerol, taking into account the stoichiometric coefficient $\nu_{i\;}$ while the yield (Equation (3)) is given by the product of conversion and selectivity.

(1) $$Glycerol\;conversion\;\left(\%\right)=\;\frac{{\left(n_{glycerol}\right)}_{in}-{\left(n_{glycerol}\right)}_{out}}{{\left(n_{glycerol}\right)}_{in}}\times 100$$

(2) $$Selectivity_{i}\;\left(\%\right)=\;\frac{{\left(n_{i}\right)}_{out}}{\nu_{i\;}\left[{\left(n_{glycerol}\right)}_{in}-{\left(n_{glycerol}\right)}_{out}\right]}\times 100$$

(3) $$Yield_{i}\;\left(\%\right)=\;\frac{Glycerol\;conversion\;\left(\%\right)\;\times \;Selectivity_{i}\;\left(\%\right)}{100}=\frac{{\left(n_{i}\right)}_{out}}{\nu_{i\;}{\left(n_{glycerol}\right)}_{in}}\times 100$$

**Figure 6:**
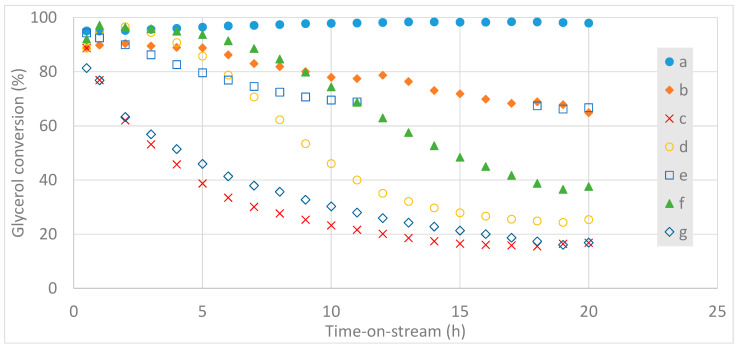
Glycerol conversion at 650 °C, feed 10 wt% glycerol in water: (**a**) La_0.3_Zr_0.7_NiO_3_; (**b**) 10 wt% Ni/(0.3La_2_O_3_–0.7ZrO_2_); (**c**) La_0.8_Sr_0.2_NiO_3_; (**d**) CeNiO_3_; (**e**) La_0.3_Ce_0.7_NiO_3_; (**f**) 10 wt% Ni/(0.3La_2_O_3_–0.7CeO_2_); (**g**) LaNiO_3_.

**Figure 7:**
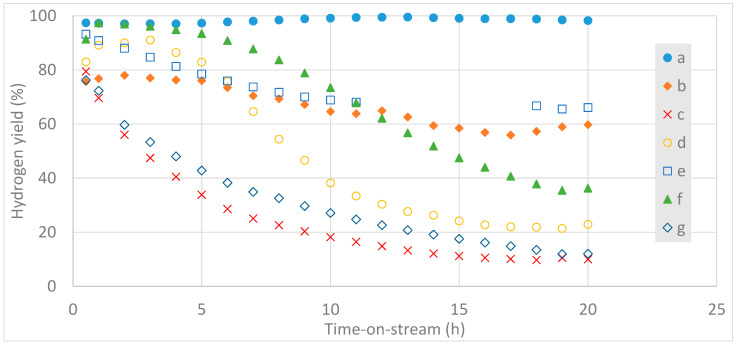
Hydrogen yield at 650 °C, feed 10 wt% glycerol in water. (**a**) La_0.3_Zr_0.7_NiO_3_; (**b**) 10 wt% Ni/(0.3La_2_O_3_–0.7ZrO_2_); (**c**) La_0.8_Sr_0.2_NiO_3_; (**d**) CeNiO_3_; (**e**) La_0.3_Ce_0.7_NiO_3_; (**f**) 10 wt% Ni/(0.3La_2_O_3_–0.7CeO_2_); (**g**) LaNiO_3_.

**Figure 8:**
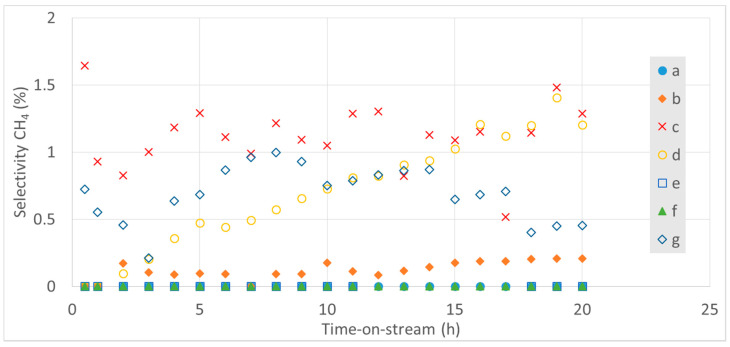
Selectivity to CH_4_ at 650 °C, feed 10 wt% glycerol in water. (**a**) La_0.3_Zr_0.7_NiO_3_; (**b**) 10 wt% Ni/(0.3La_2_O_3_–0.7ZrO_2_); (**c**) La_0.8_Sr_0.2_NiO_3_; (**d**) CeNiO_3_; (**e**) La_0.3_Ce_0.7_NiO_3_; (**f**) 10 wt% Ni/(0.3La_2_O_3_–0.7CeO_2_); (**g**) LaNiO_3_.

**Figure 9:**
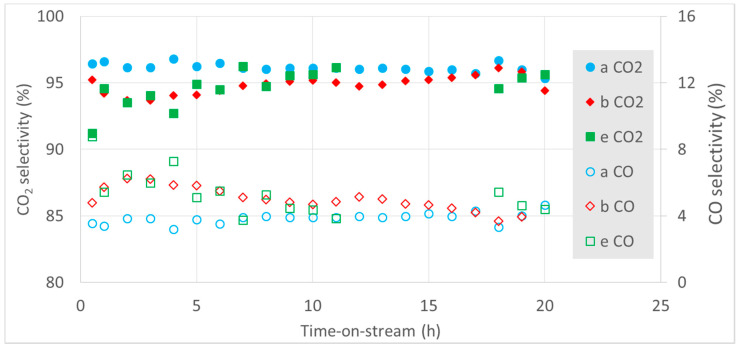
Selectivity to CO (right) and CO_2_ (left) at 650 °C, feed 10 wt% glycerol in water. (**a**) La_0.3_Zr_0.7_NiO_3_; (**b**) 10 wt% Ni/(0.3La_2_O_3_–0.7ZrO_2_); (**e**) La_0.3_Ce_0.7_NiO_3_.

The results of catalytic activity testing at 650 °C are compared for all samples reporting glycerol conversion (Figure 6), hydrogen yield (Figure 7), and selectivity to CH_4_ (Figure 8). Only a single temperature was considered, on the basis of previous investigations [49,50]. The latter is a common byproduct, which is hardly reformable except at a very high temperature (750–800 °C) over Ni catalysts, and decreases hydrogen yield. An example of the selectivity patterns for two interesting samples is also reported in Figure 9.

The selectivity to hydrogen was close to 100%, leading to a yield pattern very similar to the conversion one (Figure 6 and Figure 7). This indicates the absence of significant amounts of hydrogen containing byproducts. The carbon containing products were mainly CO_2_ (predominant) and CO, with a limited amount of CH_4_ for some catalysts only.

The less active samples were La_1−x_Sr_x_NiO_3_, which revealed initial glycerol conversion between 80 and 90%, but very rapidly deactivated to ca. 20% residual conversion after only 20 h on stream. CeNiO_3_ also demonstrated insufficient stability. The mixed formulation La_0.3_Ce_0.7_NiO_3_ showed higher initial conversion and a little better stability with time-on-stream, though still unsatisfactory. Worse long-term results were obtained with Ni impregnated on the La_2_O_3_-CeO_2_ mixed support compared to when it was added with the other precursors in mixed oxide synthesis The latter sample reached a ca. 65% plateau conversion with respect to ca. 40% for the impregnated sample.

The best results in terms of both initial conversion and, above all, optimal resistance with time-on-stream were achieved with the La_0.3_Zr_0.7_NiO_3_ sample. Also for this system, the introduction of nickel by impregnation worsened the catalytic performance, in terms of glycerol conversion. 

A similar profile is evident for hydrogen yield, given its selectivity is almost quantitative in the gas phase products. Some methane may be present and incompletely reformed under the selected reaction conditions (Figure 8). Methane can form through direct decomposition of glycerol, but more likely by methanation of CO (R2) and CO_2_ (R3), as Ni is a well-known methanation catalyst [51].

CO + 3 H_2_ ⇆ CH_4_ + H_2_O (R1)

CO_2_ + 4 H_2_ ⇆ CH_4_ + 2 H_2_O (R2)

Moreover, in this case, worse performance is exhibited by the La_1−x_Sr_x_NiO_3_ samples, leading to ca. 1% selectivity, while CeNiO_3_ gave rise to a steadily increasing methane selectivity, possibly meaning a progressive deactivation of the active phase, less able to complete the further conversion of this byproduct. Again, the best performing sample was the La_0.3_Zr_0.7_NiO_3_, showing nil selectivity to methane for the whole duration of the test.

The trends of CO and CO_2_ selectivities are reported in Figure 9 for some of the best performing samples. The predominant product was CO_2_, with selectivity always higher than 90%, while CO ranged below 10%. This is a positive feature as further hydrogen purification is carried out to remove CO and its limited selectivity implies easier removal thorugh water gas shift reaction or pressure swing adsorption. Moreover, in this case, perfectly stable data were attained for sample La_0.3_Zr_0.7_NiO_3_, while a slightly decreasing selectivity to CO with time-on-stream is shown by the other two samples.

The two most critical features identified for catalyst deactivation are, operating at high temperature, the metal sintering, and the deposition of coke. The presence of a mixed oxide precursor phase promotes a greater dispersion of Ni and a high interaction with the support. Both these features should ensure suitable resistance to sintering. On the other hand, coking is related to different aspects. In this work, support acidity, catalysing the formation of olefins and their olygomers, was tuned by selecting intrinsically basic support (La_2_O_3_, possibly added with Sr). Given its quite low melting temperature, it was mixed with ZrO_2_, a high melting point non-reducible oxide, whose acid features are in turn limited by the presence of La_2_O_3_. On the other side, CeO_2_ was used as support characterised by high oxygen mobility at the reaction temperature, in principle helping to prevent coke accumulation by oxidation of the forming nuclei of coke.

**Figure 10:**
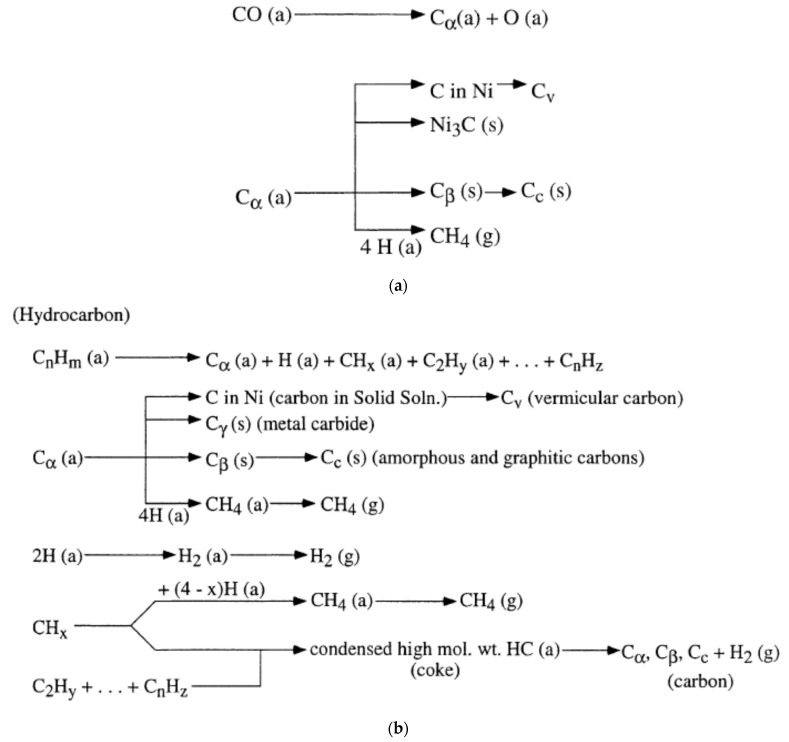
Coking mechanisms. (**a**) Formation, transformation, and gasification of carbon on nickel. (**b**) Formation and transformation of coke on metal surfaces (a, g and s refer to adsorbed, gaseous, and solid states, respectively). Reproduced with kind permission of Elsevier from [52].

The CO disproportion reaction is mainly responsible for the formation of C deposits, while the condensation of hydrocarbons, e.g., olefins, due to acidity of the support (or Lewis acidity due to the active metal itself), could lead to coke species with a different C/H ratio. The different coke formation mechanisms are sketched in Figure 10, considering the pathway from CO and from hydrocarbons’ condensation [52].

Overall, different C species are expected on the catalyst surface, with specific reference to Ni containing ones: (i) adsorbed, atomic surface carbide forming between 200 and 400 °C (C_α_); (ii) polymeric, amorphous films, or filaments, forming between 250 and 500 °C (C_β_); (iii) C filaments and nanotubes, forming between 300 and 1000 °C (C_v_); (iv) bulk NiC, forming between 150 and 250 °C (C_γ_); and (v) graphitic platelets or films, forming between 500 and 550 °C (C_c_). The different species form preferably in the indicated temperature ranges and only some metals are able to form stable carbides and nanotubes/whiskers, among which Ni is one of the most active. The different C species are also characterised by different stability in the reaction environment, as they can react with steam and H_2_ to form CO and CH_4_. For instance, C_α_ reacts with H_2_ at ca. 200 °C, C_β_ at 400 °C, and C_v_ between 400 and 600 °C. On the contrary, graphitic carbon reacts only at a much higher temperature, between 550 and 850 °C, allowing it to accumulate over the catalyst.

TGA in air was carried out by heating up to 1000 °C to determine the possible weight loss due to the combustion of C-containing species deposed over the catalyst surface; Table 2. The temperature range of C oxidation is also correlated with the stability of the coke, and thus allows one to formulate hypotheses on its nature.

All the samples showed a positive peak (weight increase) due to the oxidation of reduced Ni. Indeed, it was roughly proportional to Ni content. CeO_2_ containing samples did not reveal any weight loss attributed to coke combustion,. supporting the hypothesis that the widely known oxygen mobility and buffering properties of this oxide may be an effective prevention of coke accumulation over the catalyst. The La_1−x_Sr_x_NiO_3_ samples showed many different negative peaks, appearing atvery different temperature ranges. Being composed of non reducible oxides (except NiO), the high temperature peak cannot be attributed to oxygen loss from the support. The lowest temperature weight loss can be ascribed to adsorbed, unstructured C, while the high temperature ones to more structured C, such as the whiskers or graphitic one. By looking at the catalytic activity tests, these were the samples more rapidly deactivating, which indeed suggests the fast coke accumulation over the Ni surface and possibly over the support. The latter is also involved in water activation, so the coverage of its surface by coke hinders this part of the reaction mechanism.

On the contrary, sample La_0.3_Zr_0.7_NiO_3_ showed one weight loss feature, quite limited (−1.4 wt%) occurring at a relatively high temperature.

This result, together with the exceptional stability of this sample during the test, leads to exclude the accumulation of coke on Ni or the formation of nanotubes. It is possible that the TGA peak may correspond to some coke accumulated over some residual acidic ZrO_2_ sites, which irreversibly deactivate. Slightly greater weight loss and lower oxidation temperature were obtained for the 10 wt% Ni/(0.3La_2_O_3_–0.7ZrO_2_) sample. This can be ascribed to Ni coking (possibly with formation of nanotubes) to explain the progressive catalyst deactivation observed in Figure 6.

By comparing these results with the performance of the catalyst, taking into account the results of the structural characterization, it can be confirmed that the greater dispersion of Ni obtained by adding Ni during FP synthesis is effective in increasing its dispersion (lower reducibility) and thermal stability towards the sintering

Almost 100% conversion of glycerol was similarly reported for 15% Ni/La_2_O_3_-ZrO_2_ and 12%Ni/CeO_2_-ZrO_2_, tested under similar conditions [17]. However, most catalytic systems showed a decreasing conversion trend with time-on-stream, underlying deactivation issues mainly related to coking. Perovskite-based catalysts were also tested for this application. For instance, LaNiO_3_ tested at 650 °C led to 58 to 72% conversion, depending on the preparation method and conditions [53,54]. Moreover, when the Ni precursor was in the form of hydrotalcite, better performance and most of all stability was reported, attributing deactivation by coking to sintering of the Ni nanoparticles [17], in line with recent findings.

To sum up, the steam reforming of glycerol can be a valuable option to valorise this byproduct of biodiesel production in the same commercial compartment, i.e., the energy sector, better matching the increasing production volume. Despite the promising preliminary results in terms of activity, two main issues needed solutions: (i) the employment of noble metals in most of the best performing catalyst formulations; (ii) insufficient catalyst stability. Therefore, in the present work, we successfully focused on the application of Ni as active phase, which was already demonstrated to be active for many different substrates and is less expensive than noble metals. As for the second point, the key problem to be addressed is catalyst coking, which can be ascribed to two main issues—the acidity of the catalyst and the possible formation of carbon nanotubes due to the subsurface accumulation of carbides at the Ni/support interphase.

To cope with these pointswe have applied different strategies. On one hand, we have confirmed that the Ni dispersion and the interaction with the support is a good strategy to limit the growth of carbon over the metal particle. Therefore, we have directly incorporated Ni in mixed oxide formulations with the precursors of the support. This improved the Ni dispersion after reduction and tightened the strength of the interaction with the support. These materials were compared with samples obtained by wet impregnation of Ni over the support. TPR was selected as a tool to interpret the strength of the metal–support interaction, i.e., the higher the reduction temperature of Ni, the stronger the metal–support interaction and/or the higher the metal dispersion (in principle, improving the resistance to both sintering and coking).

**Scheme 1:**
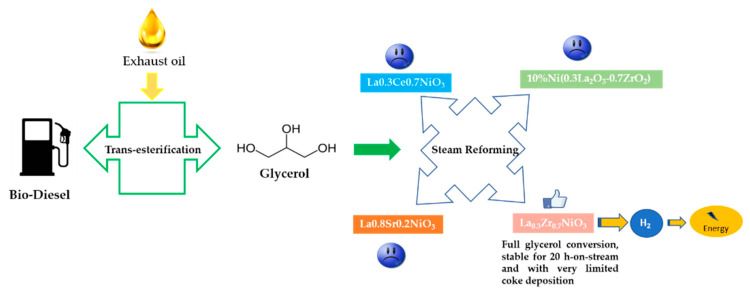
Main challenge and outcomes.

Finally, the flame pyrolysis technique adopted for the oxides synthesis was able to impart sufficient thermal resistance and to favor the Ni dispersion inside the precursor oxide material. The main challenge of this research work, the adopted strategies, and the results are summed up in Scheme 1.

## 4. Conclusions

The steam reforming of glycerol was studied as a mean to valorise a byproduct of biodiesel production for the production of hydrogen. The catalysts were designed based on Ni as active phase, supported over basic or oxygen conducting oxides. In order to favour a strong metal-support interaction, which ensures good metal dispersion and stability to sintering, mixed oxide formulations were designed with varying nominal formulation. A really stoichiometric mixed oxide was achieved only in the case of La_1−x_Sr_x_NiO_3_ catalysts, which, however, were revealed to be very poorly stable during activity testing and prone to coking. On the contrary, formulations containing CeO_2_ were not really active, but they did not accumulate coke during testing. The incorporation of Ni during the FP synthesis, even in a high amount, was beneficial to improve its dispersion and interaction strength with the support, testified by lower Ni reducibility. By contrast, the addition of Ni by impregnation led to worse performing samples, less active and more prone to coking.

Overall, the best results were obtained with catalyst La_0.3_Zr_0.7_NiO_3_, which led to full glycerol conversion, stable for 20 h on stream and with very limited coke deposition, likely covering the not-titrated most acidic sites of ZrO_2_ that, once deactivated, were completely ruled out.

## Figures and Tables

**Table 1:** Composition and textural properties of the catalysts prepared by flame spray pyrolysis (FP). SSA = specific surface area, evaluated through the Brunauer Emmet Teller model. Micropore SSA evaluated from the t-plot method.

Composition	Code	BET SSA (m^2^/g)	Micropore SSA (m^2^/g)	Crystal Size (nm)
La_0.3_Zr_0.7_NiO_3_	a	63	11	28
10 wt% Ni/(0.3La_2_O_3_–0.7ZrO_2_)	b	44	4	35
La_0.8_Sr_0.2_NiO_3_	c	56	13	17
CeNiO_3_	d	62	14	22
La_0.3_Ce_0.7_NiO_3_	e	57	14	20
10 wt% Ni/(0.3La_2_O_3_–0.7CeO_2_)	f	46	7	27
LaNiO_3_	g	53	9	14

**Table 2:** Results of TGA. ∆wt represents the normalised weight loss.

Sample	∆wt (%)	T °C	∆wt (%)	T °C	∆wt (%)	T °C	∆wt (%)	T °C	∆wt (%)	T °C
La_0.3_Zr_0.7_NiO_3_	5.7	100–400	−1.4	500–650						
10 wt% Ni/(0.3La_2_O_3_–0.7ZrO_2_)	1.0	200–400	−2.3	400–600						
La_0.8_Sr_0.2_NiO_3_	−1.5	250–300	4.9	300–500	−2.5	650–700	−1.5	700–750	−1.5	800–850
CeNiO_3_	4.9	200–400								
La_0.3_Ce_0.7_NiO_3_	5	100–400								
10 wt% Ni/(0.3La_2_O_3_–0.7CeO_2_)	1.6	150–400								
LaNiO_3_	−0.6	260–310	3.9	310–600	−1.5	600–750	−0.5	850–900	−0.3	950–1000

## Data Availability

Data is contained within the article or supplementary material.
